# Provincial Medical and Surgical Association. District Branches

**Published:** 1837-01

**Authors:** 


### PROVINCIAL MEDICAL AND SURGICAL ASSOCIATION. DISTRICT BRANCHES.

In order to increase the efficiency and interest of the Provincial Association, it has been deemed expedient, by the members resident at a distance from the midland counties, acting on the recommendation of the council, contained in the Report of 1834, relative to Sectional Meetings, to embody themselves into DISTRICT BRANCHES, and to call upon all their brethren in the different districts to unite themselves with them in furtherance of the general objects of the Institution. Besides the *Eastern Branch*, established last year, and which we are happy to say has now united itself with the Parent Association, several other district branches have been formed, and are now forming, in different parts of England. By means of these Branches, the number of the members of the Association will be greatly increased, and its utility and interest much enhanced. Indeed, it seems probable that, by the arrangements now in progress, a large proportion of the whole body of provincial practitioners will belong to it before many years are expired. We shall notice the institution of these District Branches, as they come to our knowledge.

#### *Bath District Branch.*

This Branch was instituted at a meeting of the Bath members of the General Council, held at Bath, on the 18th November. The objects in the formation of this Branch are stated to be, "to uphold the general designs of the Association, and to afford to those members who are unable to attend the Anniversary meetings of the General Association, opportunity for cultivating friendly feelings, and the intercourse of social harmony with their professional brethren, at least once a year." The organization of the Branch, the nature and scope of its proceedings, are postponed to the first Sectional meeting, which is fixed to take place at Bath on the first Thursday of June. Mr. Tudor, of Bath, was elected president for the ensuing year, and Dr. Barlow secretary.

#### *Southern District Branch.*

On Tuesday, the 29th November, a meeting of the members of the profession, residing in the counties of Hants, Wilts, Dorset, Sussex, Surrey, and Berks, was held at Southampton, for the purpose of establishing a Southern Branch. The meeting was attended by upwards of seventy gentlemen from various parts of the above-named counties, all of whom seemed only desirous of being made acquainted with the nature and objects of the proposed Branch, in order to enrol their names as members. Dr. Forbes, of Chichester, was called to the chair, and, after an excellent explanatory address by Mr. Wickham, various resolutions declaratory of the nature and objects of the Branch Association were proposed by different gentlemen, and agreed to with the greatest unanimity. The fundamental principles of the Local or Sectional Society are, 1. That its members are, *ipso facto*, members of the Parent Association, and entitled to all its privileges; and, 2. That all the public or official expenses incurred by the Branch are defrayed out of the general funds of the Association. The subordinate arrangements of the Branch Association are to be regulated exclusively by its own members. At this meeting it was determined, that the Local Society, in imitation of the general one, should have its President, Vice-Presidents, Secretaries, and Council; that it should have an annual meeting, at each of the chief towns in the district in succession, and that the papers communicated for publication by its members, after being first laid before the Local Council, should be transmitted to the General Council. Dr. Forbes, of Chichester, was elected President for the present year; Dr. Crawford, of Winchester, President Elect; W. J. Wickham, Esq. of Winchester, and Dr. Joseph Bullar, of Southampton, Secretaries; and all the members present at the Meeting, Councillors. The first general meeting of the members is to be held in the second week of June, at Winchester, under the Presidency of Dr. Crawford. Upwards of fifty new members joined the Association on the day of the Meeting; and there is little doubt that four times that number will have given in their names as members, before the general meeting in August.

---

